# A Multi-Ingredient Supplement Improves Body Re-Composition, Ovarian Aging Markers, and Reproductive Success in Young and Middle-Aged Female Mice

**DOI:** 10.3390/biom15091258

**Published:** 2025-08-30

**Authors:** Alessandra Chiarot, Mahek Minhas, Nicoletta M. de Maat, Jenny Doan, Mats I. Nilsson, Bart P. Hettinga, Mehrnoosh Faghih, Michael S. Neal, Joshua P. Nederveen, Mark A. Tarnopolsky

**Affiliations:** 1 Department of Pediatrics, McMaster University Medical Center (MUMC), Hamilton, ON L86 4L8, Canada; 2 Department of Kinesiology, McMaster University, Hamilton, ON L86 4L8, Canada; 3 Exerkine Corporation, McMaster University Medical Center (MUMC), Hamilton, ON L86 4L8, Canada; 4 Department of Obstetrics and Gynecology, McMaster University Medical Center (MUMC), Hamilton, ON L86 4L8, Canada; 5 One Fertility, Burlington, ON L7N 3T1, Canada

**Keywords:** aging, fertility, obesity, fat, muscle, nutraceuticals, antioxidants, mitochondria, oxidative stress, inflammation

## Abstract

Ovarian aging is characterized by mitochondrial dysfunction, oxidative stress, and inflammation. The development of adjunctive treatments that mitigate age-related subfertility is warranted. We examined the benefits of nutraceutical supplementation (FE; Fertility Enhancer) with mitochondrial antioxidants, anti-inflammatory agents, metabolic activators, vitamins and minerals, and amino acids on ovarian aging, metabolic activity, and reproductive success in young (Y; 6-month-old) and middle-aged (O; 11-month-old) female C57BL/6J mice. The mice were fed calorie- and macronutrient-matched diets w/wo the FE supplement for three months and harem mated twice. Daily FE supplementation promoted significant body re-composition, including loss of white adipose tissue (gWAT: −36% vs. CON, *p* < 0.001), gain of skeletal muscle (SkM: +67% vs. CON, *p* < 0.001), and improved SkM/gWAT ratio (+185% vs. CON, *p* < 0.001). Metabolic testing showed enhanced fat oxidation (+38%, *p* < 0.01) and energy expenditure (+7%, *p* = 0.051) in FE mice. Breeding and immunoblotting data demonstrated improved reproductive success (Y-CON: 44%, Y-FE: 89%, O-CON: 0%, O-FE: 18%) and a modest attenuation of ovarian aging markers in both FE groups. We surmise that a multi-ingredient supplement, such as the Fertility Enhancer, may improve body re-composition, metabolic activity, and markers of ovarian aging, thus enhancing reproductive health and fertility in females.

## 1. Introduction

Ovarian aging is defined as the progressive loss of the primordial follicle pool and a decrease in both quantity and quality of oocytes [1], resulting in fertility decline, endocrine dysfunction, and menstrual cycle abnormalities [2]. Although the ovary consists of numerous cell populations, single-cell multi-omics and transcriptomics have confirmed coordinated changes in the aging hallmarks across cell types, including mitochondrial dysfunction, oxidative stress, and inflammation [3,4]. On average, female fertility is reduced by ~6% for 25–29 years olds, ~14% for 30- to 34-year-olds, and ~31% for 35- to 39-year-olds, with a precipitous decline thereafter and complete reproductive senescence at menopause [5].

Fertility is also affected by lifestyle factors that may contribute significantly to lower pregnancy rates at advanced maternal age [6,7]. Excessive bodyweight (BW) gain is linked to overweightness/obesity, ectopic lipid deposition, and metabolic dysfunction [8], causing multiple alterations to the endocrine system and the Hypothalamic–Pituitary–Gonadal (HPG) axis [9], thus increasing the risk of subfertility and poor pregnancy outcomes. The recent discovery that muscle-derived myostatin (GDF 8) controls follicle-stimulating hormone (FSH) production also connects the musculoskeletal system to the HPG axis [10,11]. Thus, maintaining a healthy body composition index (BCI; muscle-to-fat ratio [12,13]) may be advantageous for optimizing fertility and reproductive health at advanced maternal age.

Considering that the cost of assisted reproduction technologies (ARTs) may be prohibitive from a patient perspective [14,15,16], a more defined focus on low-cost preventive measures and adjunctive therapies may strengthen overall clinical practice and enhance the affordability of fertility treatments. The utilization of nutraceutical products to support natural conception and optimize ARTs has expanded significantly in recent years. Despite this growing interest, many patients independently select supplements based on non-scientific sources, exposing themselves to formulations with unproven efficacy and inconsistent quality. As recently reviewed by others [17,18], the current marketplace remains largely unregulated, leading to substantial variability in product composition and therapeutic claims. This phenomenon underscores the urgent need for a systematic and evidence-based approach to nutraceutical use in reproductive medicine.

Currently, there is strong, scientific support for combined iron and folic acid (IFA) intake during peri-pregnancy, and the World Health Organization (WHO) has long recommended IFA supplements for maternal health and fetal development [19,20,21,22]. The inclusion of other micronutrients in so-called multiple micronutrient supplements (MMS), such as zinc; selenium; copper; iodine; and vitamins A, D, E, B_1_, B_2_, B_3_, B_6_, and B_12_, may further reduce the incidence of low birthweight and improve cost-effectiveness vs. IFAs [19,23]. Although clinical evidence is limited from high-income countries, the efficacy of MMS has been demonstrated in the UK, France, and USA previously [24,25,26]. In terms of a more targeted approach, a systematic review recently highlighted the importance of zinc, copper, and selenium for female fertility [27]. Collectively, IFAs or targeted MMS are recommended in clinical practice for improving maternal health and pregnancy outcomes.

The use of other nutraceuticals must be evaluated on a case-by-case basis and by overall scientific merit. Ideally, a nutritional strategy should aim to support a healthy body composition index and attenuate the hallmarks of ovarian aging, including mitochondrial dysfunction, oxidative damage, and inflammation [3,4]. Several meta-analyses have convincingly demonstrated that supplementation with long-chain, polyunsaturated omega-3 fatty acids (n-3 PUFAs; EPA and DHA) reduces oxidative stress [28,29] and inflammation [30,31,32] and improves risk factors associated with cardiometabolic disease [33,34], metabolic syndrome (MetS) [35,36], type 2 diabetes (T2DM) [37,38], and polycystic ovary syndrome (PCOS) [39,40,41]. Furthermore, n-3 PUFAs provide direct reproductive benefits by reducing the incidence of preterm births (PTB) [42,43,44], the leading cause of child death [45]. Thus, the multi-systemic benefits of EPA and DHA provide a compelling case for including them in routine practice.

Mitochondrial antioxidants, such as co-enzyme Q_10_ (CoQ_10_), α-lipoic acid (α-LA), and vitamin E (α-tocopherol), also have direct therapeutic relevance for fertility medicine. CoQ_10_ is an electron carrier between complexes I/II and III in the electron transport chain (ETC) of the inner mitochondrial membrane, with known benefits for overall health, ovarian aging, and reproduction [46,47,48]. Meta-analyses have previously demonstrated that CoQ_10_ supplementation improves clinical pregnancy rates, the number of retrieved oocytes, and high-quality embryo rates in women undergoing IVF [46,48]. Other meta-analytic data have shown that CoQ_10_ intake improves the reproductive hormone profile [49], glycemic control [50,51], systemic inflammation [52,53,54], oxidative stress [52,55], and blood lipid levels [56,57] in both healthy and clinical populations. Vitamin E (α-tocopherol) is another potent antioxidant found in the inner and outer mitochondrial membranes that prevents lipid peroxidation and may be regenerated by either CoQ_10_ or α-LA [58,59,60,61,62]. In terms of clinical significance, vitamin E improves systemic inflammation [58,63] and hormonal and metabolic profiles in PCOS patients [64] and is currently included in WHO’s MMS for antenatal care [19]. Lastly, α-lipoic acid is a cofactor in the mitochondrial matrix essential for oxidative metabolism and energy production [65]. Supplementation with α-LA is associated with moderate weight loss [66,67,68] and improves glycemic control [69], lipid profiles [69], and inflammation [70] in patients with metabolic disease and/or PCOS [71]. Thus, while monotherapy with either CoQ_10_, vitamin E, or α-LA is likely beneficial for reproductive health at advanced maternal age and/or in metabolic disease, a combination of these mitochondrial antioxidants provides synergism, recycling, and maintenance of the antioxidant reserve [59,62]. Other nutraceuticals that can safely enhance mitochondrial function, antioxidant defense, and cellular energy charge may also have therapeutic potential, such as creatine monohydrate (CrM) [72,73,74,75,76] and select natural polyphenols [77,78,79,80,81,82].

**Figure 1:**
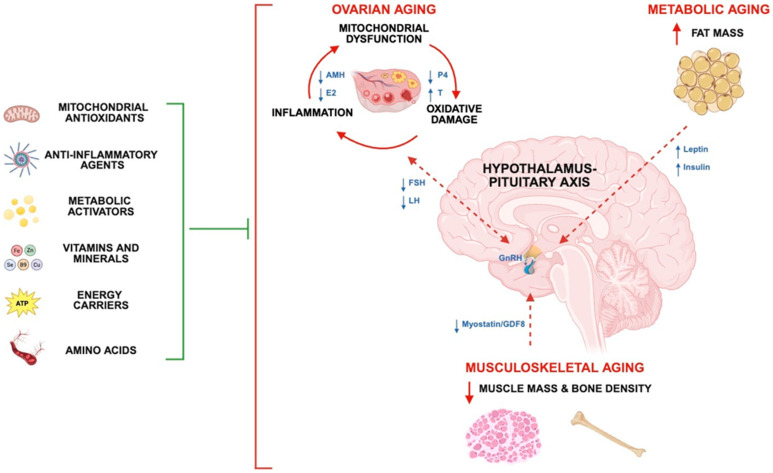
Schematic demonstrating interconnected organ systems and pathways for improving fertility and reproductive health by research-proven nutraceuticals. Human fertility and reproduction are governed by a complex interplay between the reproductive (gonads and sex hormones), neuroendocrine (HP-axis), integumentary (fat), and musculoskeletal (muscle) systems. Ovarian aging is defined as a loss of oocyte quantity and quality, with a vicious cycle of mitochondrial dysfunction, oxidative damage, and inflammation being a central component of the aging process. The concurrent loss of functional reserves of supportive organ systems contributes to metabolic impairment, HPG dysregulation, and fertility decline. An idealized, nutraceutical approach for attenuating gonadal, metabolic, and musculoskeletal aging, such as the ‘Fertility Enhancer’, is composed of mitochondrial antioxidants, anti-inflammatory agents, metabolic activators, vitamins and minerals, energy carriers, and amino acids. Dotted lines represent endocrine pathways that are impaired with aging. Blunt arrow signifies attenuation of age-related impairment (s). AMH: Anti-Müllerian hormone, P4: progesterone, E2: estradiol, T: testosterone, FSH: follicle-stimulating hormone, LH: luteinizing hormone, GDF8: growth differentiation factor 8 (Myostatin).

Considering the complex interplay between multiple organ systems in the regulation of reproductive health and fertility, we developed a unisex concept for targeting the common pathways that underlie male and female subfertility with research-proven nutraceuticals. We then designed a multi-ingredient supplement (MIS; ‘Fertility Enhancer’) with mitochondrial antioxidants, anti-inflammatory agents, metabolic activators, vitamins and minerals, energy carriers, and amino acids for attenuating gonadal, metabolic, and musculoskeletal aging (Figure 1). Next, we demonstrated proof-of-concept in a Western diet (WD)-fed mouse model and showed that the MIS protected against bodyfat gain and improved the body composition index, non-alcoholic fatty liver disease (NAFLD), ovarian inflammation, and reproductive success [83]. In this study, we aimed to explore the female fertility benefits further and demonstrate supplement efficacy in an aging mouse model under normal caloric conditions. We hypothesized that the MIS would improve body re-composition, metabolic function, markers of ovarian aging, and reproductive success in both young and middle-aged, female C57BL/6J mice. We further predicted that the body composition index (i.e., muscle-to-fat ratio) and select ovarian aging markers, such as oxidative damage and mitochondrial antioxidants, would provide the highest explanatory power for live births across age cohorts.

## 2. Methods

### 2.1. Ethics Approval

All methods and procedures were approved by McMaster University Animal Research Ethics Board on 4 May 2023, and conformed to the standards of the Canadian Council on Animal Care (AUP 20-04-17; 23-091).

### 2.2. Animals, Cohorts, and General Study Design

**Figure 2:**
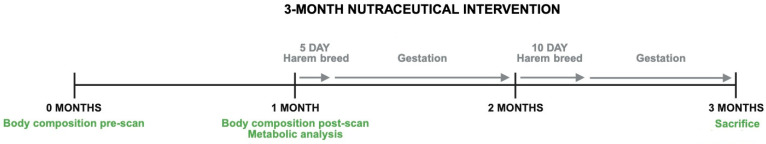
The experimental timeline of the 3-month nutraceutical intervention. Animals underwent a body composition (NMR) pre-scan at baseline (0 months) followed by one month of dietary intervention. At 1 month, the NMR post-scan and a metabolic analysis were performed, followed by five days of harem breeding. Gestation continued into the second month, after which ten days of harem breeding ensued. The study concluded at 3 months with animal sacrifice and tissue harvest.

Young male (6-month-old), young female (Y; 6-month-old), and middle-aged female (O; 11-month-old) C57BL/6J mice were obtained from the aging colony at Jackson Laboratories (Bar Harbor, MA, USA). Following five days of acclimation, the females were matched according to bodyweight and allocated into two nutraceutical interventions (i.e., control vs. ‘Fertility Enhancer’ diets) within each age cohort (Y-CON, Y-FE, O-CON, and O-FE; *n* = 15–16/group) (Table 1). Sample sizes were based on a previous study on the effects of the FE supplement on obesity-associated infertility in mice [83]. The three-month dietary intervention consisted of ad libitum food intake during peri-pregnancy with in vivo body composition scans and metabolic tests performed at baseline and after one month, followed by two consecutive breeding and gestational periods, sacrifice, and organ collection (Figure 2). Researchers were not blinded to group allocations during any stage of the experiment or data analysis.

The mice were euthanized by cervical dislocation, and reproductive organs (ovaries and testicles), white adipose tissue (WAT; gonadal), skeletal muscle (SkM; quadriceps), and livers excised, weighed, snap-frozen in liquid nitrogen, and stored at −80 °C for downstream analysis. To control for the male fertility factor, only female mice underwent the nutraceutical intervention prior to breeding. The males received the FE diet following the breeding period for exploratory purposes (i.e., body composition and select testicular aging markers; Figures S1 and S2). All mice were housed in standard microisolator cages with a 12 h light/dark cycle at 22 °C in the Central Animal Facility at McMaster University (Hamilton, ON, Canada).

### 2.3. Diets and Nutraceuticals

All animals had free access to food and water throughout the intervention, and food intakes and bodyweights were assessed twice weekly by a trained technician. Nutraceuticals were purchased from Gruppo Nutrition (Windsor, ON, Canada), and the diets were manufactured by Inotiv (Indianapolis, IN, USA). During acclimation, the animals were fed the same chow as provided by Jackson Laboratories (5K52), followed by either the control (8604) or the Fertility Enhancer (FE; TD.230256) diets for 12 weeks. As designed, CON and FE diets were matched in energy and macronutrient contents while higher in protein and less energy dense as compared to the 5K52 chow. Mitochondrial antioxidants (CoQ_10_, α-lipoic acid, and vitamin E), anti-inflammatory agents (n-3 PUFAs), metabolic activators (green coffee bean, green tea, forskolin, and beet extracts), vitamins and minerals (iron and folic acid), energy carriers (creatine monohydrate), and amino acids (L-arginine) were added to the FE diet (Table 2), as previously described [83].

### 2.4. Mating

Following four weeks of feeding, female cohorts underwent two consecutive mating and gestational periods with young, male C57BL/6J mice. As previously described [83], each female was harem bred in a 1:2 male-to-female ratio for five and ten days, respectively, followed by three weeks of gestation after each breeding period. Copulatory plugs and litters were counted at the start of the light cycle (06:00 a.m.) during breeding/gestational periods, and reproductive success was calculated with the formula [(sperm plugs/litters)*100]. Because C57BL/6J mice routinely cannibalize ~30% of their litters [83], PND-4 live litter size and survival were not obtained, and all pups were euthanized following confirmation of the litters.

### 2.5. Time-Domain NMR Whole-Body Composition

Time-domain nuclear magnetic resonance (TD-NMR) was used for assessing whole-body lean mass (LM), fat mass (FM), and lean mass/fat mass ratio (LM/FM) at baseline and following four weeks of nutraceutical intervention (Minispec LF90II, Bruker; Billerica, MA, USA). We have previously demonstrated that TD-NMR results are reflected in necropsy weights of white adipose tissue (WAT) and skeletal muscle (SM) and that either in vivo or ex vivo measures may be used as confirmative evidence of body re-composition [83].

### 2.6. Metabolic Activity Analysis

Metabolic analysis was conducted on *n* = 4 per group using the Promethion Core Metabolic and Behavioral Phenotyping System (Sable Systems International, Las Vegas, NV, USA), as previously described [84]. Food intake, physical activity (beam breaks), oxygen consumption (VO_2_), carbon dioxide production (VCO_2_), respiratory exchange ratio (RER; VCO_2_/VO_2_), and energy expenditure data (kcal/h) were collected every 15 min for 48 h. Beam breaks were converted into meters traveled (i.e., pedmeters), which represents movements that are active, that are directional, and that cross more than one beam. Fatty acid and carbohydrate oxidation rates were calculated using [(1.70 × VO_2_) − (1.69× VCO_2_)] and [(4.58 × VCO_2_) − (3.23 × VO_2_)], respectively.

### 2.7. Ovarian Immunoblotting

RIPA lysis buffer (ThermoFisher Scientific, Burlington, ON, Canada), supplemented with protease and phosphatase inhibitor cocktail (ThermoFisher Scientific), was added to ovarian tissue and homogenized using the Fast Prep automatic homogenizer (MP Biomedicals, Solon, OH, USA). The homogenate was centrifuged at 12,000× *g* for 20 min at 4 °C, and the supernatant was collected to measure total protein content of each sample using the BCA Protein Assay Kit (ThermoFisher Scientific) according to the manufacturer’s instructions. All samples were prepared at a constant protein concentration in Laemmli buffer (ThermoFisher Scientific), heated at 37 °C (for OXPHOS) or 95 °C for 5 min, and loaded in 4–20% Criterion TGX Stain-Free Gels (Bio-Rad Laboratories, Mississauga, ON, Canada) for gel electrophoresis. Gels were imaged using the ChemiDoc MP Imaging System (Bio-Rad Laboratories); then, protein samples were transferred to PVDF membrane (Bio-Rad Laboratories) and imaged again. Membranes were placed in 5% BSA (Sigma Aldrich, Oakville, ON, Canada) blocking solution (in 1 × TBST) for 1 h, then incubated in primary antibodies overnight at 4 °C while rocking. Primary antibodies included total OXPHOS rodent WB antibody cocktail (Abcam, Waltham, MA, USA), anti-4 hydroxynonenal (4-HNE) antibody (Abcam), SOD1 polyclonal antibody (Proteintech Group, Rosemont, IL, USA), SOD2 polyclonal antibody (Proteintech Group), NF-κB p65 (D14E12) XP^®^ rabbit mAb (Cell Signaling Technology, Boston, MA, USA), phospho-NF-κB p65 (Ser536) (93H1) rabbit mAb (Cell Signaling Technology), and phospho-NF-κB p65 (Ser468) recombinant antibody (Proteintech Group) and were all prepared in 5% BSA in 1 × TBST from stock. The following day, blots were incubated in secondary antibodies (ThermoFischer Scientific) of the corresponding primary antibodies in a 1:20,000 dilution in BSA for 2 h. Blots were washed (3 × 5 min) in 1 × TBST then placed in Clarity Max Western ECL substrate (Bio-Rad Laboratories) to be imaged. Optical densities (OD) of each band were quantified using Image Lab 6.1 (Bio-Rad Laboratories) software and normalized to their respective stain-free blots.

### 2.8. Ovarian Quality Index

A composite score was generated from the immunoblotting data using five recognized ovarian aging markers, including mitochondrial content (OXPHOS; CII and CIII), antioxidant defense (SOD2), oxidative stress (lipid peroxidation; 4-HNE), and inflammation (NFκB activation; phosphoS536/tot NFκB). The samples were ranked from lowest (or worst: 1) to highest (or best: 44), followed by calculation of the mean ovarian quality index ((CII_rank_ + CIII_rank_ + SOD2_rank_ + 4HNE_rank_ + phosphoS536/tot NFκB_rank_)/5).

### 2.9. Statistical Analyses

All data were tested for normality and homogeneity of variance by Shapiro–Wilk and Levene’s tests, respectively; 2 × 2 factorial ANOVAs (age × diet) and 2 × 2 repeated measures ANOVAs (age × diet × time) were used for analyzing main effects and interactions, followed by Fisher’s LSD post hoc tests to determine group differences (Statistica v. 12, Statsoft Inc., Tulsa, OK, USA). Specifically, all endpoint data were analyzed by 2 × 2 ANOVAs (i.e., organ weights, reproductive outcomes, metabolic activity, and ovarian aging markers), while pre-to-post changes in anthropometry and in vivo body composition were analyzed by 2 × 2 repeated measures ANOVAs. Priority comparisons were dietary effects within each age group (i.e., Y-CON vs. Y-FE and O-CON vs. O-FE, respectively) and secondarily treatment differences between age groups (i.e., diet × age interactions). Statistical outliers were removed according to the empirical rule (i.e., 95% of values in a normal distribution fall within two standard deviations of the mean).

To determine the strongest predictors of reproductive success, simple and multiple linear regression analyses were run on relevant independent variables (i.e., anthropometry, organ weights, and markers of ovarian aging) and live births (dependent variable). Dummy coding was used for categorical variables. Weakening of the overall model, negligible correlation with the outcome, non-casual correlation with the outcome (nonsense), and redundancy/collinearity with other predictors were considered as exclusion criteria from the final model.

All tables include exact *p*-values for ANOVA main effects and interactions. Between-group differences, as indicated by LSD post hoc tests, are denoted by alphabetical letters (*p* ≤ 0.05). Main effects and interactions are shown with symbols in figures, including age (✣), diet (†), time (§), age*diet (⦾), age*time (‡), diet*time (Ω), and/or age*diet*time (◆) (*p* ≤ 0.05). Significant LSD post hoc tests between priority groups are denoted by star symbols in figures, such as (* *p* ≤ 0.05), (** *p* ≤ 0.01), and (*** *p* ≤ 0.001). Trending *p*-values are reported if biologically relevant. All data are presented as means ± SEM.

## 3. Results

### 3.1. Food Intakes

Daily food consumption and caloric intakes were significantly higher in both young and middle-aged FE groups vs. age-matched controls (Table 3). Because CON and FE diets were matched in energy and macronutrient contents, we surmise that the Fertility Enhancer stimulated appetite and food consumption across age cohorts (*p* < 0.0001).

### 3.2. Metabolic Activity

**Figure 3:**
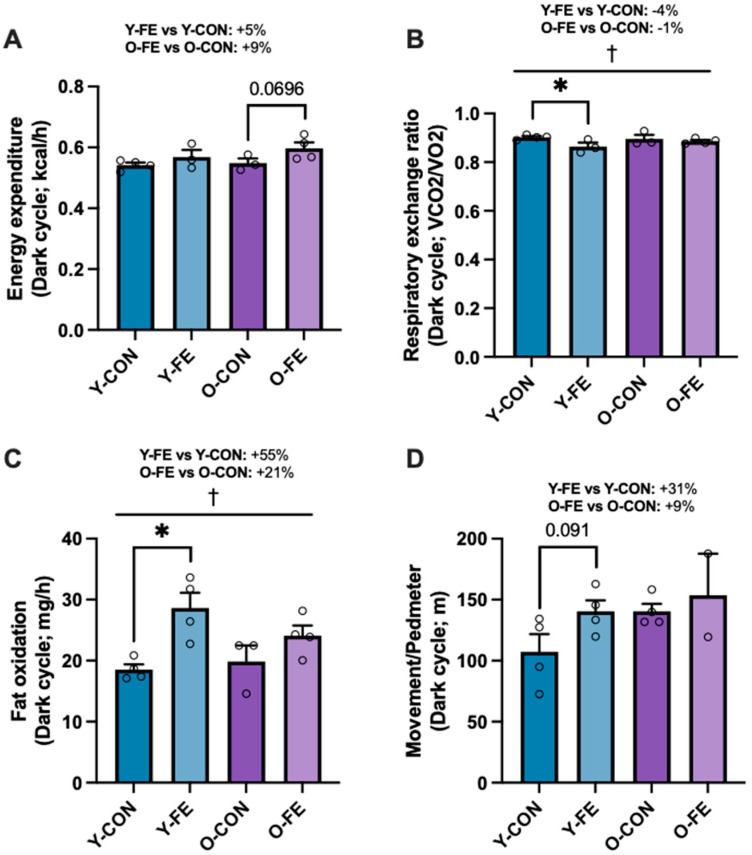
In vivo metabolic testing after one month of dietary intervention in young and middle-aged female mice. (**A**) Energy expenditure, (**B**) the respiratory exchange ratio (RER), (**C**) fat oxidation, and (**D**) movement/pedmeter in the control and FE groups during the dark cycle period (18:00 –06:00). Data are presented as means ± SEM. Sample sizes were *n* = 2–4 per group following the removal of outliers. † 2 × 2 factorial ANOVA main effect of diet (*p* ≤ 0.05). Significant LSD post hoc tests between priority groups are denoted by the star symbol (* *p* ≤ 0.05). Trending *p*-values are shown if biologically relevant.

Next, we assessed in vivo metabolic activity and found that energy expenditure was higher across age cohorts in FE-treated mice (Figure 3A; *p* = 0.051). FE groups had significantly lower respiratory exchange ratios (Figure 3B; *p* < 0.05), had higher fatty acid oxidation rates (Figure 3C; *p* < 0.01), and tended to move more (Figure 3D; *p* = 0.13) vs. age-matched controls (Tables S1 and S2). Thus, daily FE intake improved metabolic activity in both young and middle-aged females under normal caloric conditions, as previously demonstrated in a high-calorie Western diet model [83].

### 3.3. Anthropometry and Body Composition

**Figure 4:**
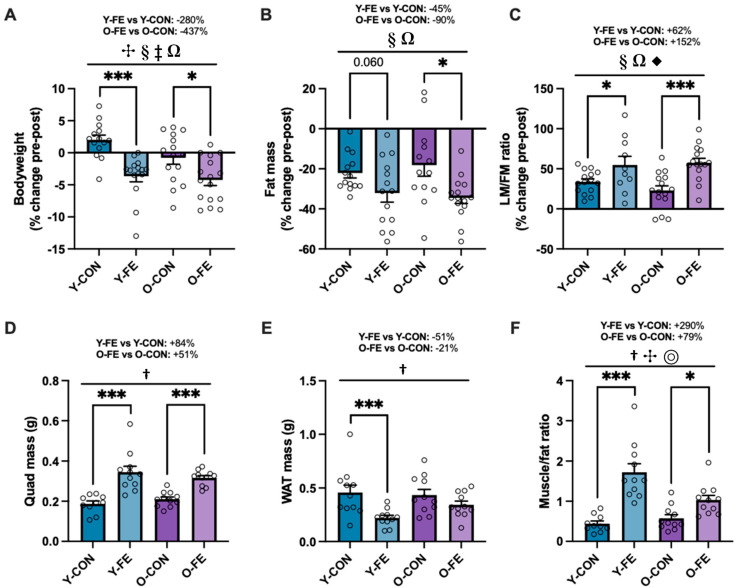
In vivo anthropometry, body composition (NMR), and ex vivo organ weights. (**A**–**C**) Percent changes from baseline to post-intervention in anthropometry (bodyweight) and NMR results (fat mass and LM/FM ratio). (**D**–**F**) Post-intervention organ weights for quadriceps muscle, gonadal WAT, and muscle/fat ratio. Data are presented as means ± SEM. Sample sizes were *n* = 11–16 per group following removal of outliers. Significant main effects from 2 × 2 RM ANOVAs or 2 × 2 factorial ANOVAs are denoted by symbols, including age (✣), diet (†), and/or time (§) (*p* ≤ 0.05). Significant ANOVA interactions are also denoted by symbols, including age*diet (⦾), age*time (‡), diet*time (Ω), and/or age*diet*time (◆) (*p* ≤ 0.05). Significant LSD post hoc tests between priority groups are denoted by star symbols (* *p* ≤ 0.05) and (*** *p* ≤ 0.001). Trending *p*-values are shown if biologically relevant.

Despite higher daily caloric intakes in both FE groups (Table 3), bodyweights were significantly reduced following one month of FE supplementation (Figure 4A and Table S3), attributed to improved lipid oxidation (Figure 3C) and whole-body fat loss (Figure 4B). Considering that both CON and FE diets contained more protein and less calories vs. the acclimation diet, changes in whole-body composition were expected across all groups; however, the adaptations were superior in the FE-treated cohorts (Figure 4C and Table S3). These findings were corroborated ex vivo by excising and weighing the most relevant fat pad to reproduction (gonadal WAT) and a major hindlimb muscle group (quadriceps) following breeding and gestation (Figure 4D–F and Table S4). These treatment benefits were also consistent in males following one month of FE supplementation (Figure S1). In a previous study, we conducted pair-feeding experiments and demonstrated that up to 30–40% of anti-obesity effects may be driven by central/appetite-suppressive effects by select nutraceuticals in the FE supplement [83]. However, because appetite was increased in both FE groups herein, the weight loss was likely driven by other factors, such as enhanced thermogenesis (i.e., WAT browning), causing significant body re-composition and an improved muscle-to-fat ratio.

### 3.4. Reproduction

As expected, there were fewer litters produced by middle-aged (O) vs. young (Y) females (main effect of age; *p* < 0.01), while copulatory behavior (sperm plugs) was largely unaffected by aging and supplementation (Table 4; Cumulative Results). Cumulative litter counts were quantitatively higher in FE-supplemented vs. control mice across age cohorts, but this did not reach statistical significance (main effect of diet; *p* = 0.10). However, considering that reproductive success was more than doubled in FE vs. control groups, we surmise that FE supplementation improved fertility and reproductive health in both young and middle-aged female mice.

### 3.5. Ovarian Aging Markers

Next, to determine the dietary effects on ovarian aging markers, we analyzed mitochondrial OXPHOS (complexes I–V), oxidative damage (4-HNE), antioxidant defense (SOD1 and SOD2), and inflammation (NFκB activation) in ovarian tissue by immunoblotting (Table 5). While FE-treated animals exhibited consistent improvements in most markers, the benefits were relatively modest (i.e., ~20% vs. controls) and more pronounced in the young cohort. Specifically, CII (+16%; *p* = 0.037), CIII (+22%; *p* = 0.056), SOD2 (+23%; *p* = 0.11), 4-HNE (−11%; *p* = 0.20), and phospho/total NFκB (−22%; *p* = 0.064) improved/tended to improve in FE vs. CON groups. Dietary effects were less apparent for CI, CIV, CV, and SOD1 (Table S5).

### 3.6. Ovarian Quality Index

**Figure 5:**
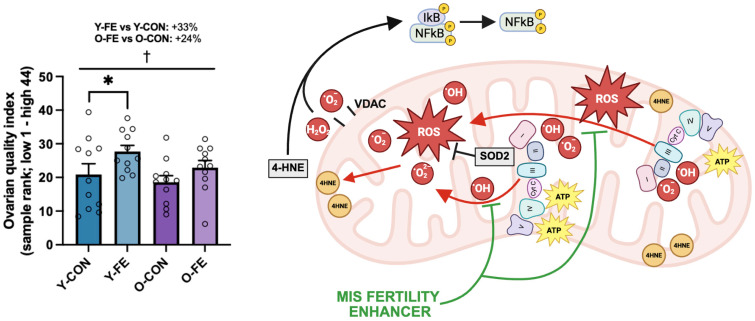
The ovarian quality index. A composite score (ovarian quality index) was generated using sample ranks of CII, CIII, SOD2, 4-HNE, and phospho/total NFκB immunoblotting results ((CII_rank_ + CIII_rank_ + SOD2_rank_ + 4HNE_rank_ + phosphoS536/tot NFκB_rank_)/5) (see Section 2). Data are presented as means ± SEM. The total sample size was *n* = 44 (11 per group). † diet effect (*p* ≤ 0.05). Significant LSD post hoc effects are denoted by the star symbol (* *p* ≤ 0.05).

Based on the immunoblotting findings, a composite score was generated using CII, CIII, SOD2, 4-HNE, and phospho/total NFκB sample ranks, suggestive of the FE supplement improving overall ovarian quality across age groups (Figure 5; +29% vs. CON; *p* = 0.022).

### 3.7. Female Fertility Predictors

To elucidate the underlying factors of female fertility and reproductive success, we conducted regression analyses of potential predictors (independent variables) of live births (dependent variable), including anthropometry (bodyweight), organ mass (SkM and gWAT), and markers of ovarian aging (OXPHOS, mitochondrial antioxidants, oxidative damage, and inflammation). The ex vivo muscle/fat ratio was a stronger predictor of live births vs. SkM and gWAT independently, while either organ was a better predictor than bodyweight (Table 6). Interestingly, mitochondrial antioxidants (SOD2) and oxidative damage (4-HNE) were stronger predictors of live births vs. OXPHOS (CII and CIII) and inflammation (phospho/total NFκB). Furthermore, the ovarian quality index did not supersede SOD2 in predictive strength, suggesting that mitochondrial antioxidants may be major determinants of ovarian quality and reproductive success. Based on these data, a full prediction model was generated from the ex vivo muscle/fat ratio, SOD2, and 4-HNE, which provided the highest explanatory power and statistical significance [F(3,39) = 8.886, *p* < 0.00013, R = 0.64, R^2^ = 0.41]. All three factors added predictive power and were non-redundant (i.e., no collinearity); however, only the muscle/fat ratio (*p* = 0.006) and SOD2 (*p* = 0.001) were statistically significant predictors in the full model, while 4-HNE was not (*p* = 0.12).

## 4. Discussion

Herein, we have demonstrated the proof-of-concept of a multi-ingredient supplement (MIS; Fertility Enhancer, FE) with mitochondrial antioxidants, anti-inflammatory agents, metabolic activators, vitamins and minerals, energy carriers, and amino acids for attenuating age-related subfertility. By targeting multiple organ systems and pathways that contribute to reproductive health, the FE supplement improved body composition, ovarian aging markers, and reproductive success in both young and middle-aged female C57BL/6J mice. Our findings also suggest that the body composition index (i.e., muscle-to-fat ratio) and select ovarian aging markers (i.e., mitochondrial antioxidants and oxidative damage) provide the highest explanatory power for reproductive success in this model.

Recent advances in reproductive medicine have elucidated the driving mechanisms of ovarian aging and the integral role(s) of interconnected organ systems (i.e., HPG-muscle and fat axes) in the regulation of fertility and reproductive function. Single-cell multi-omics and transcriptomics have confirmed coordinated changes in the aging hallmarks across ovarian cell types, including mitochondrial dysfunction, oxidative stress, and inflammation [3,4]. Specifically, Jin et al. [4] generated a high-resolution single-nuclei multi-omic atlas of human ovarian tissue and found that deregulated mTOR signaling and oxidative phosphorylation are signature hallmarks of ovarian aging and congruent between cell populations. Ongaro et al. recently demonstrated a direct link between SkM and the HPG axis [10], showing that myostatin is a critical regulator of FSH synthesis and fertility. It is well-known that SkM declines from the age of 25 onwards [85], concurrent with body fat accumulation and ectopic lipid deposition, all of which may contribute to metabolic dysfunction and alterations to the neuroendocrine system, circadian clock genes, and the HPG axis [9,86,87,88]. Our findings indicate that select ovarian aging markers (i.e., SOD2 and 4-HNE) and the SkM/WAT ratio (i.e., body composition index) independently predict female fertility, while a combination of all variables provides the highest explanatory power for reproductive success. We therefore conclude that adjunctive therapies should aim to promote body re-composition and attenuate the hallmarks of ovarian aging to optimize female fertility benefits.

The use of multi-ingredient supplements for targeting signaling networks, negative feedback loops, and/or interconnected organ systems in the treatment of aging- and lifestyle-associated conditions and inherited diseases is not a new concept. We have previously shown the utility of this approach for treating sarcopenia [13,89,90,91], obesity [12,92], NAFLD [93], radiation exposure [94], neuromuscular disease [95], and primary mitochondrial disease [96,97]. Recently, we also demonstrated that select nutraceuticals targeting the hallmarks of ovarian, metabolic, and musculoskeletal aging may mitigate a range of obesity-associated comorbidities, including WAT accumulation, NAFLD, and fertility impairment [83]. The results of the current study extend these findings by showing efficacy in an aging mouse model under normal caloric conditions.

We observed moderate but consistent improvements across ovarian aging markers in FE vs. control groups (~20%), with a significant main effect on the composite ovarian quality score. Mitigation of the central components of somatic and germ cell aging in ovarian tissue is a novel finding. Although monotherapy with mitochondrial antioxidants has metabolic [63,70] and reproductive benefits [46,48,53,54], a combination of redox couples, such as CoQ_10,_ α-lipoic acid, or vitamin E, adds synergism, recycling, and the maintenance of the antioxidant reserve [60,62]. This ‘mitochondrial cocktail’ is currently in use for treating mitochondrial disorders [96,97], and the addition of CrM may potentiate its benefits by enhancing cellular bioenergetics [97,98]. Plant extracts, such as green tea, green coffee bean, and forskolin, contain a spectrum of polyphenols that exert inhibitory effects on anabolic and growth signaling (i.e., mTOR) while stimulating ‘longevity pathways’ (i.e., AMPK and PGC-1 alpha) [99,100,101,102], with the potential to attenuate the hallmarks of ovarian aging. Other ingredients in the FE supplement with proven anti-inflammatory and antioxidant benefits include n-3 polyunsaturated fatty acids (n-3 PUFAs; EPA and DHA) [28,29,30,31,32], which independently also reduce the risk of preterm births [42,43,44].

Furthermore, daily FE intake enhanced energy expenditure and fat oxidation in both young and middle-aged cohorts, thereby promoting significant body re-composition under normal caloric conditions, consistent with our previous findings in a diet-induced obesity model [83]. These superior adaptations were not driven by central effects (appetite suppression), indicative of select nutraceuticals inducing favorable metabolic adaptations by WAT browning, as previously described [83,92]. Supplements containing caffeine and/or polyphenols are well-known to induce weight loss by thermogenic, lipolytic, and/or appetite suppressant effects [103,104]. Concurrent with WAT loss, FE-treated animals exhibited significantly higher muscle mass vs. controls (+51–84%) following the treatment. Several compounds in the FE supplement have anabolic potential. CrM has well-documented benefits for SkM and performance [75] and is being investigated in obstetric and neonatal medicine for preventing preterm birth [73,74,76,105]. L-arginine may also enhance SkM gains and/or sexual function by improving blood flow (nitric oxide production) [106], and meta-analytic data suggest that it prevents maternal pre-eclampsia and high blood pressure [107,108]. While zinc, selenium, copper, folic acid, iron, and vitamins B_12_ and D_3_ are unlikely to promote body re-composition, multiple micronutrient supplements have strong scientific support for optimizing fertility and pregnancy outcomes [19], especially in micronutrient deficiency states [23,109].

There are some weaknesses of the current study that should be addressed. Although multi-ingredient supplements provide more benefits than drawbacks in the treatment of complex, multi-systemic conditions, it is not possible to determine the individual contribution(s) of each component in the Fertility Enhancer. Each ingredient was carefully chosen for its safety and proven benefits on human fertility, pregnancy outcomes, and/or metabolism. Importantly, the efficacy of this formulation has been demonstrated in two separate preclinical models, including aging-associated subfertility (current study) and obesity-associated subfertility [83]. Furthermore, while fertility is a couple concept, we opted to control for the male contribution to reproductive success in this study. Preliminary data indicate that FE supplementation improves body re-composition, antioxidant defense, and key regulators of the cell cycle in the testicular tissue of male mice (Figures S1 and S2), although sperm function has not been assessed. Lastly, there is uncertainty in age equivalence between animals and humans. Based on Dutta et al. [110], we surmise that the young (7–8 months) and middle-aged (12–13 months) female cohorts were equivalent to ~24–28 and ~41–45 human years, respectively. Thus, we believe that our findings are relevant for a broad range of ages attempting pregnancy, including those in their reproductive peak and those experiencing significant fertility decline.

## 5. Conclusions

Several nutraceuticals, such as mitochondrial antioxidants, omega-3 fatty acids, and certain vitamins and minerals, have demonstrated therapeutic potential to improve spontaneous pregnancy, gamete quality, embryo development, and implantation rates when appropriately formulated and dosed in humans. In this study, we provided pre-clinical evidence that a combination of mitochondrial antioxidants (CoQ10, α-lipoic acid, and vitamin E), anti-inflammatory agents (n-3 PUFAs), metabolic activators (green coffee, green tea, forskolin, and beetroot extracts), vitamins and minerals (iron and folic acid), energy carriers (creatine monohydrate), and amino acids (L-arginine) may improve ovarian quality, body composition, and reproductive success. We surmise that an ‘idealized’ multi-ingredient supplement, such as the Fertility Enhancer, holds significant promise as an adjunctive therapy in fertility management, while future clinical trials are necessary to determine optimal dosing strategies, safety and efficacy profiles, and clinical utility in both males and females. A double-blind, placebo-controlled RCT has been registered to assess the fertility benefits of the current nutraceutical formulation (NCT06091969).

## Figures and Tables

**Table 1:** Experimental cohorts, sample sizes, and diets.

	Age		*n*-Size	Acclimation Diet	Intervention Diet
	Start (Months)	End (Months)			
**Y-CON**	6.2	9.2	15	LabDiet 5K52 (JAX)	8604 (Inotiv)
**Y-FE**	6.2	9.2	15	LabDiet 5K52 (JAX)	TD.230256 (Inotiv)
**O-CON**	11.2	14.2	16	LabDiet 5K52 (JAX)	8604 (Inotiv)
**O-FE**	11.2	14.2	16	LabDiet 5K52 (JAX)	TD.230256 (Inotiv)

Y-CON and O-CON denote young and old C57BL/6J control mice, respectively, fed a standard intervention diet (Inotiv; 8604). Y-FE and O-FE represent young and middle-aged mice fed the Fertility Enhancer (FE) diet (Inotiv; TD.230256). All animals were acclimated on LabDiet 5K52 (JAX) prior to the intervention.

**Table 2:** Macronutrient, energy, and nutraceutical profiles of control and FE diets.

	Control (8604)	Fertility Enhancer (TD.230256)	Manufacturer (Purity)
**Macronutrients and energy**			
kcal/g	3.00	3.00	Inotiv
Carbohydrate (%kcal)	54.0	49.4	Inotiv
Protein (%kcal)	32.0	30.2	Inotiv
Fat (%kcal)	14.0	20.5	Inotiv
**Mitochondrial antioxidants**			
CoQ10 (ubiquinone; g/kg)	-	2.50	MTC (98–101%)
Vitamin E (α-tocopherol; IU/kg)	120	1155	Inotiv
α-Lipoic acid (g/kg)	-	1.00	Alzchem (98%)
**Anti-inflammatory agents**			
n-3 oil (g/kg)	-	28.0	NutraSea (EPA and DHA)
**Metabolic activators**			
Green coffee extract (g/kg)	-	2.50	AFS (CGA 45%, caffeine 40%)
Green tea extract (g/kg)	-	0.75	ENI (EGCG 50%, caffeine < 3%)
Forskolin extract (g/kg)	-	0.13	Sabinsa (40%)
Beet root extract (g/kg)	-	10.0	Enovate (100%)
**Vitamins and minerals**			
Iron/ferric citrate (mg/kg)	300	700	Inotiv
Folic acid (mg/kg)	3.00	8.00	Inotiv
Zinc (mg/kg)	80.0	80.0	Inotiv
Selenium (mg/kg)	0.34	0.34	Inotiv
Copper (mg/kg)	25.0	25.0	Inotiv
Vitamin B12 (mg/kg)	0.05	0.05	Inotiv
Vitamin D3 (IU/g)	2.40	2.40	Inotiv
**Energy carriers**			
Creatine monohydrate (g/kg)	-	30.0	Alzchem (99–100%)
**Amino acids**			
L-arginine (g/kg)	15.0	20.0	Inotiv

Ingredient profiles of the control and FE diets including macronutrients, mitochondrial antioxidants, anti-inflammatory agents, metabolic activators, vitamins and minerals, energy carriers, and amino acids.

**Table 3:** Daily food and energy intakes.

	Food and Energy Intakes	
	g/d	kcal/d
**Y-CON**	3.85 ± 0.07 ^a^	11.53 ± 0.22 ^a^
**Y-FE**	4.17 ± 0.15 ^b^	12.52 ± 0.45 ^b^
**O-CON**	3.77 ± 0.06 ^a^	11.31 ± 0.17 ^a^
**O-FE**	4.26 ± 0.11 ^b^	12.78 ± 0.32 ^b^
**Main Effects**	**2 × 2 ANOVA**	**2 × 2 ANOVA**
**Age (A)**	0.96398	0.95148
**Diet (D)**	**0.00002**	**0.00001**
**Interactions**		
A*D	0.36969	0.36378

FE = Fertility Enhancer group, CON = control group. Food and energy intakes were recorded twice weekly for the entirety of the study. Data are presented as mean ± SEM. Statistically significant group differences are denoted by different letters (*p* ≤ 0.05).

**Table 4:** Mating behavior and reproductive outcomes.

	5-Day Mating Period			10-Day Mating Period			Cumulative Results		
	Plugs	Litters	Reproductive Success (%)	Plugs	Litters	Reproductive Success (%)	Plugs	Litters	Reproductive Success (%)
**Y-CON**	3.0 ^ab^	2.0 ^ab^	66.7	6.0	2.0	33.3	9.0	4.0 ^ab^	44.4
**Y-FE**	6.0 ^ab^	5.0 ^a^	83.3	3.0	3.0	100.0	9.0	8.0 ^a^	88.9
**O-CON**	2.0 ^a^	0.0 ^b^	0.0	5.0	0.0	0.0	7.0	0.0 ^b^	0.0
**O-FE**	8.0 ^b^	0.0 ^b^	0.0	3.0	2.0	66.7	11.0	2.0 ^b^	18.2
**Main Effects**	**2 × 2 ANOVA**			**2 × 2 ANOVA**			**2 × 2 ANOVA**		
**Age (A)**	0.91335	**0.00292**	NA	0.93797	0.28014	NA	0.82145	**0.00571**	NA
**Diet (D)**	**0.01475**	0.18828	NA	0.14439	0.22359	NA	0.45292	0.10121	NA
**Interactions**									
A*D	0.44739	0.18828	NA	0.81547	0.64873	NA	0.452921	0.54925	NA

C57BL/6J mice underwent two separate 5- and 10-day harem-breeding periods in a 1:2 male-to-female ratio. Sperm plugs and litters were counted at the beginning of the light cycle each morning (6:00 a.m.). Reproductive success was calculated with the formula [(sperm plugs/litters)*100]. Sample sizes were *n* = 15–16 females per group. Data are presented as means ± SEM. Statistically significant group differences are denoted by different letters (*p* ≤ 0.05).

**Table 5:** Immunoblotting results of ovarian aging markers.

	Mitochondrial		Antioxidant	Oxidative Damage	Inflammation		
	Complex II	Complex III	SOD2	4HNE	pNFkb (Ser536)	tNFkb	p/tNFkb
**Y-CON**	0.020 ± 0.002 ^a^	0.021 ± 0.002	0.028 ± 0.004	0.041 ± 0.003	0.025 ± 0.010	0.023 ± 0.008	1.351 ± 0.650
**Y-FE**	0.026 ± 0.002 ^a^	0.026 ± 0.002	0.035 ± 0.004	0.035 ± 0.004	0.022 ± 0.007	0.30 ± 0.008	0.859 ± 0.367
**O-CON**	0.021 ± 0.002 ^a^	0.021 ± 0.002	0.026 ± 0.004	0.046 ± 0.003	0.025 ± 0.011	0.025 ± 0.008	1.133 ± 0.566
**O-FE**	0.024 ± 0.002 ^a^	0.023 ± 0.001	0.031 ± 0.004	0.043 ± 0.004	0.021 ± 0.014	0.025 ± 0.008	1.055 ± 0.818
**Main Effects**	**2 × 2** **ANOVA**	**2 × 2** **ANOVA**	**2 × 2 ANOVA**	**2 × 2** **ANOVA**	**2 × 2** **ANOVA**	**2 × 2 ANOVA**	**2 × 2 ANOVA**
**Age (A)**	0.94530	0.43540	0.42328	0.07822	0.84741	0.78773	0.73208
**Diet (D)**	**0.03715**	0.05595	0.11030	0.20410	0.21210	0.22683	0.06406
**Interactions**							
A*D	0.49087	0.36999	0.90435	0.80935	0.59630	0.37531	0.79310

Results are presented as mean optical densities ± SEM. Sample sizes for analyzed blots were 9–11 per group after removing outliers. Representative immunoblots are shown in the Supplementary Materials. Statistically significant group differences are denoted by different letters (*p* ≤ 0.05).

**Table 6:** Correlational analysis of fertility predictors in female mice.

Fertility Predictors	Linear Regression Results		
**Anthropometrics**			
Bodyweight	*r* = −0.170	*r*^2^ = 0.029	*p* = 0.270
**Ex vivo body composition**			
Muscle (quad)	*r* = 0.2414	*r*^2^ = 0.058	*p* = 0.119
Fat (gWAT)	*r* = −0.3390	*r*^2^ = 0.115	***p* = 0.026**
Muscle/fat ratio	*r* = 0.3702	*r*^2^ = 0.137	***p* = 0.015**
**Ovarian aging markers**			
Complex II	*r* = 0.1830	*r*^2^ = 0.033	*p* = 0.235
Complex III	*r* = 0.2816	*r*^2^ = 0.079	*p* = 0.064
SOD2	*r* = 0.4866	*r*^2^ = 0.237	***p* = 0.001**
4HNE	*r* = −0.3013	*r*^2^ = 0.091	***p* = 0.047**
p/tNFkb	*r* = −0.0917	*r*^2^ = 0.008	*p* = 0.554
Ovarian quality index	*r* = 0.3296	*r*^2^ = 0.109	***p* = 0.029**
**Best multiple regression model**	R = 0.637	R^2^ = 0.406	***p* = 0.00013**
Muscle/fat ratio	***p* = 0.00633**		
SOD2	***p* = 0.00109**		
4HNE	*p* = 0.11814		

Pearson correlation coefficients (*r*), coefficient of determination (*r^2^*), and *p*-values are reported for each individual fertility-related predictor. Anthropometric, body composition, and select ovarian aging markers were assessed for their association with fertility outcomes. The best multiple regression model was muscle-to-fat ratio, SOD2 and 4HNE, providing the highest explanatory power for live births. Significant correlations have bolded *p*-values (*p* ≤ 0.05).

## Data Availability

The original contributions presented in this study are included in the article. Further inquiries can be directed to the corresponding author.

## Supplementary Materials

The following supporting information can be downloaded at https://www.mdpi.com/article/10.3390/biom15091258/s1. Table S1: Metabolic activity outcomes. Table S2: Physical activity outcomes. Table S3: In vivo anthropometry and body composition (NMR). Table S4: Ex vivo muscle mass, fat mass, and muscle-to-fat ratio. Table S5: Additional immunoblotting results of ovarian aging markers. Figure S1: Male anthropometry and in vivo body composition following FE supplementation. Figure S2. Male fertility markers following FE supplementation.
