# Supplementary material for: A Multi-Ingredient Supplement Improves Body Re-Composition, Ovarian Aging Markers, and Reproductive Success in Young and Middle-Aged Female Mice
Source: Biomolecules. 2025 Aug 30;15(9):1258. doi: 10.3390/biom15091258 (PMC12466991; doi:10.3390/biom15091258)

Supplementary Information

Table S1: Metabolic activity outcomes.

Metabolic activity was assessed using an indirect calorimetry system (Sable Systems International). Measurements were taken during both light and dark cycles and include respiratory exchange ratio (RER), energy expenditure, carbohydrate oxidation, and fat oxidation. Data are presented as means ± SEM.

	**Energy expenditure (EE; kcal/h)**			**Respiratory exchange ratio (RER)**			**Carbohydrate oxidation (mg/h)**			**Fat oxidation (mg/h)**		
	**Daily**	**Light Cycle**	**Dark Cycle**	**Daily**	**Light Cycle**	**Dark Cycle**	**Daily**	**Light Cycle**	**Dark Cycle**	**Daily**	**Light Cycle**	**Dark Cycle**
**Y-CON**	0.51 ± 0.00	0.48 ± 0.01	0.54 ± 0.01	0.87 ± 0.00	0.84 ± 0.01	0.90 ± 0.00	81.40 ± 2.30	62.08 ± 5.88	100.79 ± 3.09	22.68 ± 0.84	25.20 ± 1.00	18.52 ± 0.85
**Y-FE**	0.52 ± 0.02	0.48 ± 0.01	0.57 ± 0.02	0.85 ± 0.01	0.84 ± 0.00	0.86 ± 0.02	74.53 ± 6.64	62.47 ± 1.70	86.58 ± 11.50	27.50 ± 1.37	26.42 ± 0.98	28.62 ± 2.48
**O-CON**	0.51 ± 0.01	0.48 ± 0.02	0.55 ± 0.01	0.87 ± 0.01	0.85 ± 0.01	0.90 ± 0.02	81.13 ± 8.33	63.15 ± 6.05	99.26 ± 10.54	22.62 ± 1.95	25.39 ± 1.45	19.85 ± 2.64
**O-FE**	0.54 ± 0.02	0.48 ± 0.03	0.60 ± 0.02	0.87 ± 0.01	0.86 ± 0.01	0.89 ± 0.01	85.87 ± 2.23	69.73 ± 4.77	101.74 ± 2.91	24.06 ± 1.06	24.12 ± 1.17	24.09 ± 1.65
**Main Effects**	**2 X 2 ANOVA**			**2 X 2 ANOVA**			**2 X 2 ANOVA**			**2 X 2 ANOVA**		
**Age (A)**	0.579780	0.806197	0.316271	0.276978	0.338244	0.474051	0.279500	0.441587	0.352849	0.331790	0.388313	0.479646
**Diet (D)**	0.132400	0.642116	0.051207	0.315707	0.489367	**0.044040**	0.829562	0.516911	0.421263	**0.022136**	0.981012	**0.007135**
**Interactions**												
**A*D**	0.508303	0.622779	0.524871	0.259114	0.686319	0.203945	0.258591	0.564261	0.259918	0.138190	0.308394	0.196604

Table S2: Physical activity outcomes.
Physical activity was measured using automated beam-break sensors (X, Y, and Z axes). Total movement counts were recorded over light and dark cycles. Data are presented as means ± SEM.

	Pedmeter (m)			X-beam breaks			Y-beam breaks			Z-beam breaks		
	**Daily**	**Light Cycle**	**Dark Cycle**	**Daily**	**Light Cycle**	**Dark Cycle**	**Daily**	**Light Cycle**	**Dark Cycle**	**Daily**	**Light Cycle**	**Dark Cycle**
**Y-CON**	144.8 ± 13.3	37.5 ± 5.5	107.2 ± 14.4	15,997 ± 1,342	3,966 ± 154	12,189 ± 1,849	20,128 ± 739	6,114 ± 1,053	14,486 ± 1,692	15,486 ± 1,131	5,905 ± 484	9,788 ± 1,130
**Y-FE**	183.2 ± 15.7	42.8 ± 7.6	140.3 ± 9.1	16,115 ± 447	4,197 ± 382	12,137 ± 249	21,519 ± 312	5,549 ± 547	16,376 ± 549	14,288 ± 1,835	4,396 ± 633	10,029 ± 1,744
**O-CON**	171.7 ± 7.3	31.3 ± 6.3	140.3 ± 6.2	15,865 ± 854	3,532 ± 410	12,590 ± 1,152	19,788 ± 654	4,692 ± 601	15,393 ± 898	14,736 ± 2,216	5,452 ± 1,198	9,442 ± 1,087
**O-FE**	228.6 ± 49.9	33.4 ± 2.8	153.5 ± 34.1	18,067 ± 1,603	3,545 ± 195	13,472 ± 1,185	21,444 ± 1,225	4,424 ± 265	17,324 ± 1,495	18,507 ± 1,589	5,193 ± 996	13,551 ± 967
**Main Effects**		**2 X 2 ANOVA**			**2 X 2 ANOVA**			**2 X 2 ANOVA**			**2 X 2 ANOVA**	
**Age (A)**	0.422900	0.213626	0.129450	0.821272	0.185966	0.767511	0.732970	0.097829	0.431415	0.337938	0.847549	0.234331
**Diet (D)**	0.157506	0.769794	0.130230	0.997670	0.812323	0.946569	0.097295	0.501451	0.122157	0.473325	0.332386	0.111994
**Interactions**												
**A*D**	0.444636	0.659597	0.494464	0.443128	0.744296	0.445511	0.803073	0.773961	0.985957	0.178416	0.488419	0.153229

Table S3: *In vivo* anthropometry and body composition (NMR).
Whole-body MRI was used to non-invasively assess bodyweight, lean mass, fat mass, and body fat percentage. Measurements were performed prior to and post dietary intervention. Data are presented as means ± SEM

	**Bodyweight (BW; g)**		**Fat mass (FM; g)**		**Lean mass (LM; g)**		**LM/FM ratio**	
	**Pre**	**Post**	**Pre**	**Post**	**Pre**	**Post**	**Pre**	**Post**
**Y-CON**	25.31 ± 0.42	25.81 ± 0.46	5.64 ± 0.48	4.38 ± 0.38	17.70 ± 0.20	18.40 ± 0.22	3.26 ± 0.23	4.37 ± 0.34
**Y-FE**	25.15 ± 0.55	24.19 ± 0.41	6.15 ± 0.61	4.13 ± 0.30	17.83 ± 0.29	17.54 ± 0.30	2.82 ± 0.33	4.05 ± 0.35
**O-CON**	26.77 ± 0.58	26.49 ± 0.41	6.47 ± 0.58	4.98 ± 0.42	18.35 ± 0.22	18.69 ± 0.20	2.83 ± 0.21	3.49 ± 0.20
**O-FE**	27.24 ± 0.63	26.04 ± 0.49	7.08 ± 0.59	4.58 ± 0.39	18.37 ± 0.36	18.79 ± 0.27	2.73 ± 0.19	4.37 ± 0.31
**Main Effects**	**2 X 2 RM ANOVA**		**2 X 2 RM ANOVA**		**2 X 2 RM ANOVA**		**2 X 2 RM ANOVA**	
**Age (A)**	**0.002982**		0.100437		**0.008215**		0.259806	
**Diet (D)**	0.372502		0.775792		0.542431		0.977273	
**Time (T)**	**0.000274**		**0.000000**		**0.001899**		**0.000000**	
**Interactions**								
**A*D**	0.361153		0.975618		0.395694		0.110858	
**T*A**	**0.045781**		0.398786		0.329209		0.932609	
**T*D**	**0.000011**		**0.038824**		**0.013640**		**0.008398**	
**T*A*D**	0.283339		0.763273		**0.004120**		**0.031704**	

Table S4: *Ex vivo* muscle mass, fat mass, and muscle-to-fat ratio.

Ex vivo measurements of muscle mass, fat mass, and muscle-to-fat ratio were obtained following sacrifice. Tissues were dissected and weighed to determine absolute wet weights. Data are presented as means ± SEM.

	**Quad Mass (g)**	**WAT Mass (g)**	**Quad/WAT**
**Y-CON**	0.187	0.459	0.442
**Y-FE**	0.345	0.221	1.723
**O-CON**	0.210	0.435	0.575
**O-FE**	0.317	0.343	1.030
**Main Effects**	**2 X 2 ANOVA**	**2 X 2 ANOVA**	**2 X 2 ANOVA**
**Age (A)**	0.885559	0.479442	**0.045291**
**Diet (D)**	**0.000000**	**0.000434**	**0.000000**
**Interactions**			
**A*D**	0.174039	0.066871	**0.004062**

Table S5: Additional immunoblotting results of ovarian aging markers.

Total protein content normalized to gel for immunoblotting experiments of additional targets. Data are presented as means ± SEM.

	Mitochondrial			Antioxidant	Inflammation		
	**Complex I**	**Complex IV**	**Complex V**	**SOD1**	**pNFkb (Ser468)**	**tNFkb**	**p/tNFkb**
**Y-CON**	0.021 ± 0.003	0.20 ± 0.004	0.021 ± 0.003	0.031 ± 0.004	0.001 ± 0.000	0.023 ± 0.008	0.038 ± 0.018
**Y-FE**	0.027 ± 0.003	0.027 ± 0.004	0.027 ± 0.003	0.030 ± 0.004	0.001 ± 0.000	0.030 ± 0.008	0.027 ± 0.013
**O-CON**	0.024 ± 0.003	0.023 ± 0.003	0.021 ± 0.003	0.030 ± 0.005	0.001 ± 0.001	0.025 ± 0.008	0.036 ± 0.024
**O-FE**	0.021 ± 0.003	0.022 ± 0.002	0.018 ± 0.002	0.029 ± 0.005	0.001 ± 0.001	0.025 ± 0.008	0.034 ± 0.021
**Main Effects**	**2 X 2 ANOVA**	**2 X 2 ANOVA**	**2 X 2 ANOVA**	**2 X 2 ANOVA**	**2 X 2 ANOVA**	**2 X 2 ANOVA**	**2 X 2 ANOVA**
**Age (A)**	0.618354	0.751352	0.149394	0.703302	0.756502	0.523595	0.718466
**Diet (D)**	0.648025	0.345695	0.664755	0.795027	0.734077	0.161924	0.283796
**Interactions**							
**A*D**	0.119607	0.292702	0.153537	0.958050	0.912409	0.160169	0.476956

Figure S1. Male anthropometry and body composition following FE supplementation.
(A-D) NMR results for bodyweight, fat mass, lean mass, and LM/FM ratio after 30 days of FE diet post breeding and gestation periods. Data are presented as means ± SEM. Significant LSD post-hoc effects are denoted by star symbols (***$p \leq 0.001$).

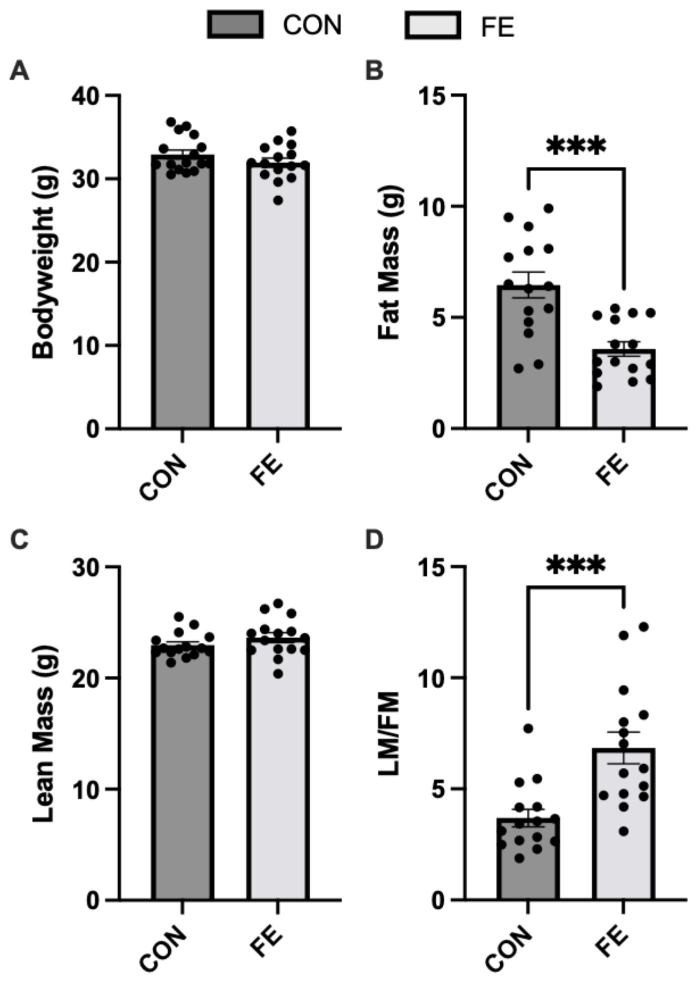

Figure S2. Male fertility markers following FE supplementation.

Top: qPCR gene expression of cell cycle regulators p16 and p21 in testicular tissue of control and FE supplemented male mice. Bottom: Protein expression (OD) of mitochondrial antioxidant SOD2 and cytosolic antioxidant SOD1 in testicular tissue of control and FE supplemented male groups. Data are presented as means ± SEMs. Exact *p*-values shown.

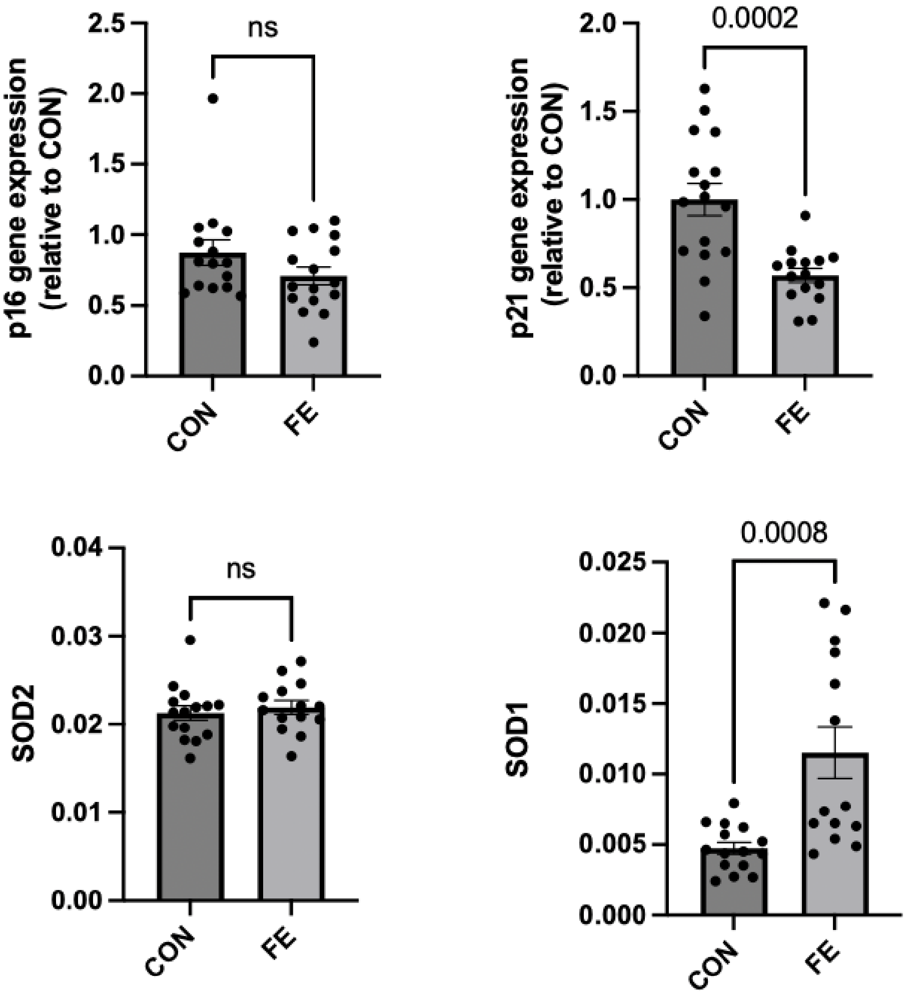

Supplement: Supplementary file 1 [file biomolecules-15-01258-s001.zip › biomolecules-3721170-supplementary.pdf]
